# Detection of *Toxoplasma gondii* in chicken and soil of chicken farms in Nanjing region, China

**DOI:** 10.1186/s40249-017-0277-3

**Published:** 2017-05-09

**Authors:** Xin-Chao Liu, Yu He, Deng-Ge Han, Zhen-Chao Zhang, Ke Li, Shuai Wang, Li-Xin Xu, Ruo-Feng Yan, Xiang-Rui Li

**Affiliations:** College of Veterinary Medicine, Nanjing Agricultural University, Nanjing, Jiangsu 210095 People’s Republic of China

**Keywords:** *Toxoplasma gondii*, Chickens farms, Soil, Chicken

## Abstract

**Background:**

Soil is increasingly recognized as an important source in the transmission of *Toxoplasma gondii* (*T. gondii*). The aim of this study was to investigate the presence of *T. gondii* in the soil and to grasp the relationships between the contamination of soil and chicken infections.

**Methods:**

PCR method based on *T. gondii*-conserved gene internal transcribed spacer 1 (ITS-1) as target gene and ELISA method (sGRA8-ELISA) using the recombinant protein of shortened GRA8 gene of *T. gondii* as antigen were developed and applied. From April 2013 to March 2014, a total of 700 soil samples were collected at various sites located in thirty farms categorized as free range farm and scale farm in Nanjing, Jiangsu, China, in different seasons. Additionally, a total of 350 sera of chickens were collected from free range farms to determine the presence of antibodies against *T. gondii* using sGRA8-ELISA.

**Results:**

The serological results showed that, antibodies were found in 194 of 250 (67.14%) samples from farms with *T. gondii* positive in soil and 41 of 100 samples from farms with *T. gondii* negative in soil (41.00%) (*P* < 0.01). The PCR detection of soil samples showed that, 7 (2.0%) of 350 samples collected from feeding zone in free range farms were found positive of *T. gondii*, whereas no sample was positive in scale farms. In the seasonal detections, *T. gondii* was found in 6 (3.33%) samples collected in autumn and 1 (0.56%) collected in winter.

**Conclusions:**

The results indicated that the contamination of *T. gondii* in soil in the free range farms was higher than that in the scale farms and seroprevalence of *T. gondii* in chickens in the farm with soil contamination was higher than that with no soil contamination. The soil contamination might be an effective indicator of *T. gondii* infection in chickens.

**Electronic supplementary material:**

The online version of this article (doi:10.1186/s40249-017-0277-3) contains supplementary material, which is available to authorized users.

## Multilingual abstracts

Please see Additional file 1 for translations of the abstract into the five official working languages of the United Nations.

## Background

Toxoplasmosis is a zoonotic disease caused by the obligate intracellular parasite *T. gondii*. Toxoplasmosis can cause severe neurologic, ocular, and systemic diseases in neonates and individuals with weakened immune system [1]. *T. gondii* infections are widely prevalent in human beings and animals worldwide [2, 3]. In China, many investigations have been done to estimate the prevalence of *T. gondii* infection in swine [4], poultry [5] and in the shellfish and fish [6]. It has been accepted that postnatal infections in humans are acquired by ingesting one of the two persistent stages of *T. gondii*, i.e. tissue cysts in meat or viscera of many animals and oocysts [7]. It was estimated that *Toxoplasma* caused 8% of hospitalizations and 24% of deaths resulting from foodborne illnesses in the United States [8]. So far no foodborne toxoplasmosis has been reported in China. But findings from recent studies indicate that *T. gondii* encysts in muscle more efficiently than in the brain [2], making chicken, one of the main meat source in China, a potentially significant source of infection.

The result of serological surveys in China indicates a high prevalence of infection in chickens [5, 9]. In our laboratory, a total of 1 173 free range chicken serum samples from 13 provinces/municipalities were tested for *T. gondii* circulating antigens (TCA) and antibodies (TCAb), respectively. The results showed that 199 (16.97%) were positive for TCA, 226 (19.27%) were positive for TCAb, 69 (5.88%) were positive for both TCA and TCAb, and the total seropositive rate was found in 356 (30.35%) out of 1173 samples. Although it was lower than that of other reports [10–12], all of the detected provinces were found of positive samples of *T. gondii* [13]. In brief, *T. gondii* in chickens is a large threat to poultry industry as well as chickens consumers.

The oocysts of *T. gondii* are produced by its final host, the domestic cat or wild felines [2, 14]. Cats can excrete millions of oocysts into soil after ingesting only one bradyzoite or one tissue cyst, and the sporulated oocysts of *T. gondii* can remain infective in soils for 18 months under various temperatures [15]. So soil is a major source of infection of *T. gondii* for both animals and humans.

Until now, a few studies have been conducted worldwide to determine the status of soil contamination with *T. gondii* by molecular methods [16, 17]. And the results indicated that the soils from gardens [18], pig farms [19], public schools [20] and public parks [21] were all contaminated with *T. gondii* oocysts in a large numbers. However, there were no reports about the soil contamination of *T. gondii* oocysts in chicken farms in China. So in this study, soil contamination with *T. gondii* in different types of chicken farms in China were investigated using PCR method and the relationship between the status of soil contamination and the infection of *T. gondii* in chickens were evaluated using the established ELISA method.

## Methods

### The parasites

*T. gondii* strain RH (type I) used in the present study is virulent strain for mice. The strain RH of human origin was stored in liquid nitrogen in the Laboratory of Veterinary Molecular and Immunological Parasitology, Nanjing Agricultural University, P.R. China. Initially, the strain was maintained by intraperitoneal passage (twice weekly) in mice. Tachyzoites were then obtained from peritoneal washings in mice inoculated. The tachyzoites were washed by centrifugation using phosphate buffered saline (PBS) and were finally resuspended in PBS.

### Establishment of the ELISA method

#### Serum samples

A total of 50 broiler chickens (*Gallus domesticus*) were obtained from a commercial farm in Nanjing City of Jiangsu Province, P. R. China. Twenty chickens at 14 days old were infected intraperitoneally with tachyzoites (10^7^) of RH strain of *T. gondii* and the sera were obtained from hearts at 7, 14, 21, 28, 45, 60, 75, 90, 130 days post-infection(DPI). The negative sera were obtained from the other 30 blank chickens at 14 days old. According to Yanming Sun’ report, the standard sGRA8 positive serum was generated [22]. Finally, the serum was collected and stored at −20 °C until use [22].

The serum against 7 *Eimeria* coccidian strains (*Eimeria.tenella, Eimeria.maxima, Eimeria.mitis, Eimeria.acervulina, Eimeria.praecox, Eimeria.necatrix, Eimeria.brunetti*) were stored in our laboratory. Serum against newcastle disease virus (NDV) and infectious bursal disease virus (IBDV) were graciously provided by professor Li yin and Wang yongshan, institute of Veterinary Research, Jiangsu Academy of Agricultural Sciences, PR China. The *Escherichia coli* infected chicken serum was gifted from professor Wang chuanqing, Henan Agricultural University, PR China.

#### sGRA8-ELISA method

The sGRA8 protein was stored in our laboratory. Sera diluted at 1:10 were added to 96-well polystyrene microstate ELISA plates coated with 0.1 mL (170 ng) of the sGRA8 antigen and incubated for 2 h at 37 °C. The plates were then washed three times with 0.1 mL phosphate-buffer-saline-Tween (PBST) and diluted horseradish peroxidase (HRP)-conjugated goat anti-chicken IgG (Sigma) (1:6 000) was added. Peroxidase activity was revealed by adding 0.1 mL of tetramethylbenzidine (TMB) substrate buffer solution for 10 min at room temperature. The reaction was then stopped immediately and OD value was measured at 450 nm using microplate spectrophotometer (Bio-Rad Laboratories, Hercules, CA, USA).

#### The cut-off between positive and negative serum

Thirty negative sera were detected by the sGRA8-ELISA, and the cut-off between positive and negative serum was calculated from the average of the sGRA8-negative serum plus 3 standard deviations (SD) of the mean.

- The cut-off = the average of negative serum + 3SD
- Negative control: sample OD450 < cut-off;
- Positive control: sample OD450 ≥ cut-off;

#### Sensitivity and Specificity of the sGRA8-ELISA

The standard sGRA8 positive serum was diluted from 1:10 to 1:5 242 880. The ELISA was conducted according to the above method.

According to the method above, the infected sera against 7 strains of *Eimeria* coccidian, NDV, IBDV and *Escherichia Coli* were detected by sGRA8-ELISA. Meanwhile the standard sGRA8 positive serum and negative serum were detected as controls respectively.

#### Coincidence rate of sGRA8-ELISA method

The chickens sera collected from artificial infection with *T. gondii* from day 7 to day 130 were detected by the method established above. The standard sGRA8 positive serum and negative serum were detected simultaneously.

### Development of PCR method for soil detection

The PCR method based on *T. gondii*-conserved gene internal transcribed spacer 1 (ITS-1) as target gene for soil detection was conducted according to previously reported [6]. To standardize the sensitivity, diluted *T. gondii* tachyzoites at the concentration of 1 × 10^5^, 1 × 10^4^, 1 × 10^3^, 5 × 10^2^, 1 × 10^2^, 5 × 10^1^, 1 × 10^1^, 1 × 10^0^ were mixed respectively with 0.5 g blank soils. The negative control with only blank soil was set. Then the total DNA of the mixture and negative control were extracted by the commercial E.Z.N.A TM Soil DNA Kit (OMEGA, USA) according to the manufacturer’s instructions and was used as template for the PCR.

The PCR products were then loaded onto 1% agarose gels (Sigma–Aldrich, St. Louis, MO, USA). The resulting DNA fragments were visualized by the Gel Doc XR System and analyzed using Quantity One 4.6.3 software (Bio-Rad, Hercules, CA, USA). The size of the amplified PCR products was estimated by comparison with the DL2000 DNA Marker (TaKaRa, Dalian, China).

### Collection of soil and chicken samples for detection

#### Soil samples

From April 2013 to March 2014, 700 soil samples were collected from thirty farms, including 15 free range farms and 15 scale farms, in Nanjing, Jiangsu, China. In free range farms, the facilities were categorized into feeding zone and motion zone. Feeding zone is the rest area or the house for chickens. Motion zone is the distances around the feeding zone for 10 m. Five soil samples were taken from feeding zone and motion zone, respectively at each sampling time, and the total number of soil samples collected in free range farms were 350. In scale farms, no distinction was drawn between feeding zone and motion zone, and at each sampling time 10 soil samples were collected around the water place and coops, whereas 350 samples were collected from scale farms.

Besides that, the 700 soil samples were collected in different seasons. In spring, 160 samples were collected, and 180 samples were collected respectively in summer, autumn and winter. Finally, each of the seven hundred samples taken from the surface layer of the ground was about 10 g – 15 g, and prepared for further examinations (Table 1).

**Table 1:** Detection of *Toxoplasma gondii* in soil samples

The time of collected	District	Type of farms	NO. of farms	NO.of samples	Positive number
2013.04	Liuhe	Free range	1	10	0
		Scale	1	10	0
	Jiangning	Free range	1	10	0
		Scale	1	10	0
	Pukou	Free range	1	10	0
		Scale	1	10	0
	Lishui	Free range	1	10	0
		Scale	1	10	0
2013.05	Liuhe	Free range	1	10	0
		Scale	1	10	0
	Jiangning	Free range	1	10	0
		Scale	1	10	0
2013.06	Liuhe	Free range	1	10	0
		Scale	1	10	0
	Pukou	Free range	1	10	0
		Scale	1	10	0
2013.07	Liuhe	Free range	1	10	0
		Scale	1	10	0
2013.08	Liuhe	Free range	1	10	0
		Scale	1	10	0
	Jiangning	Free range	1	10	0
		Scale	1	10	0
	Pukou	Free range	1	10	0
		Scale	1	10	0
	Lishui	Free range	1	10	0
		Scale	1	10	0
	Gaochun	Free range	1	10	0
		Scale	1	10	0
2013.09	Liuhe	Free range	1	10	0
		Scale	1	10	0
	Gaochun	Free range	1	10	0
		Scale	1	10	0
	Gaochun	Free range	1	10	0
		Scale	1	10	0
2013.10	Liuhe	Free range	1	10	0
		Scale	1	10	0
	Jiangning	Free range	1	10	0
		Scale	1	10	0
2013.11	Liuhe	Free range	1	10	2
		Scale	1	10	0
	Pukou	Free range	1	10	0
		Scale	1	10	0
	Jiangning	Free range	1	10	0
		Scale	1	10	0
	Lishui	Free range	1	10	1
		Scale	1	10	0
	Gaochun	Free range	1	10	1
		Scale	1	10	0
2013.12	Liuhe	Free range	1	10	1
		Scale	1	10	0
	Pukou	Free range	1	10	1
		Scale	1	10	0
2014.01	Liuhe	Free range	1	10	0
		Scale	1	10	0
	Pukou	Free range	1	10	0
		Scale	1	10	0
	Lishui	Free range	1	10	1
		Scale	1	10	0
2014.02	Liuhe	Free range	1	10	0
		Scale	1	10	0
	Pukou	Free range	1	10	0
		Scale	1	10	0
	Gaochun	Free range	1	10	0
		Scale	1	10	0
	Lishui	Free range	1	10	0
		Scale	1	10	0
2014.03	Liuhe	Free range	1	10	0
		Scale	1	10	0
	Pukou	Free range	1	10	0
		Scale	1	10	0

#### Serum samples of chicken detected

The chicken from five free range farms with positive soil samples and 2 with negative soil samples were selected to collect serum samples. In each farm, 50 blood samples were collected randomly. Serum was separated from the collected blood by centrifugation at 5 000 × g for 5 min and stored at −20 °C until analysis.

### Detection of samples

#### Detection of *T. gondii* in soil samples by PCR method

All the 700 soil samples were detected by the PCR method established above. Positive amplicons produced in the detection were purified and sequenced using the ABI 377 automated DNA sequencer (BigDye TerminatorChemistry) employing the same primers as used in the PCR assay to confirm the identity of the products by Shanghai Invitrogen Biotech (Shanghai, China).

#### Detection of *T. gondii* in chickens by ELISA

Serum samples were detected by sGRA8-ELISA developed above together with the standard sGRA8 positive serum and negative serum.

## Results

### The establishment of the methods

#### The cut-off between positive and negative serum of IgG level of sGRA8-ELISA method

The average OD450 of negative sera was 0.206 and the standard deviation (SD) was 0.028. So the cut-off was 0.290. If sample OD450 ≥ 0.290, it was determined as positive. If sample OD450 < 0.290, it was determined as negative.

#### The sensitivity and specificity of sGRA8-ELISA method

In the ELISA sensitivity experiment, the standard sGRA8 positive serum diluted to 1:81920 was still detected as positive.

In the ELISA specificity experiment, only standard sGRA8 positive serum presented positive OD value. No positive OD value was observed in any control serum samples.

#### The coincidence rate of sGRA8-ELISA method

The result obtained from the detection showed that within 45 days after infection the coincidence rate of sGRA8-ELISA was 100%,and until to the 130th day the coincidence rate reduced to 80%. The detail results were indicated in Table 2.

**Table 2:** Detection of chicken serum infected with *Toxoplasma gondii* by sGRA8-ELISA

No.	The time of blood collection								
	7d	14d	21d	28d	45d	60d	75d	90d	130d
1	0.582	1.432	1.206	0.806	0.576	0.577	0.735	1.583	0.184
2	1.303	0.913	0.706	1.278	0.546	0.444	0.189	0.837	0.447
3	0.372	1.125	0.427	0.953	0.736	0.230	1.132	0.574	0.190
4	0.882	0.548	1.099	0.766	1.021	0.326	1.340	1.008	0.256
5	0.335	1.300	0.622	0.566	1.004	0.504	1.313	0.122	0.346
6	1.534	0.429	1.293	1.366	1.015	0.339	1.367	0.346	1.082
7	0.783	0.354	1.502	1.588	0.779	0.682	1.566	0.406	0.171
8	0.876	0.815	1.206	1.602	1.108	0.561	0.254	0.226	0.706
9	1.129	1.534	1.146	1.043	0.954	0.931	0.727	0.344	0.329
10	1.603	1.064	0.905	0.843	0.928	1.312	0.699	0.391	0.311
11	0.860	0.554	1.079	1.008	0.330	1.202	0.852	0.816	0.307
12	0.669	1.106	1.099	0.852	0.220	0.370	0.810	0.837	0.298
13	0.517	1.069	1.147	1.561	0.835	0.996	0.868	0.519	0.372
14	0.536	1.156	1.082	1.056	0.902	0.551	0.831	1.234	0.542
15	1.084	0.879	1.321	1.140	0.768	1.123	0.744	0.415	0.602
16	0.900	0.871	1.743	0.420	1.058	0.502	0.801	0.662	0.560
17	1.373	0.619	0.722	1.266	0.867	0.900	1.571	0.285	0.851
18	.0787	1.261	0.728	1.053	0.376	0.797	1.483	1.117	0.422
19	0.552	1.009	1.661	1.183	0.159	1.167	1.200	0.519	0.266
20	0.394	1 .161	1.464	1.277	0.798	1.094	0.811	0.385	0.790
Positive percent	100%	100%	100%	100%	100%	95%	90%	85%	80%

Each value in the table was the average of two rapeatition

#### The sensitivity of PCR method

**Fig. 1:**
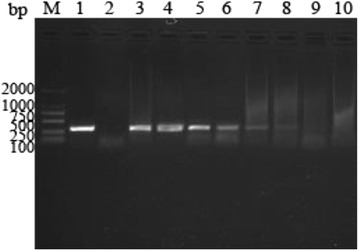
The sensitivity of PCR to detect the *Toxoplasma* tachyzoites DNA in 0.5 g soil. Lane M: molecular size marker; lane 1: positive control; lane 2: negative control; lanes 3–10: 1 × 10^5^, 1 × 10^4^, 1 × 10^3^, 5 × 10^2^, 1 × 10^2^, 5 × 10^1^, 1 × 10^1^, 1 × 10^0^ of *Toxoplasma* tachyzoites in 0.5 g soil, respectively

Positive products were found in all dilutions except 1 × 10^1^ and 1 × 10^0^ of *T. gondii* tachyzoites in 0.5 g soil (Fig. 1). The DNA sequence analysis indicated that the product was consistent with the ITS-1sequence of *T. gondii*.

### Detection of *T. gondii* in soil samples

#### The overall ratio of positive in soil samples

As shown in Table 1, 7 samples (1%) were detected positive for *T. gondii* by PCR. Sequence analysis showed that the amplicons shared 100 identities with the ITS-1 sequence of the *T. gondii* RH strain (GenBankTM accession number AY259044.1).

#### Comparison of soil contamination for *T. gondii* between free range farms and scale farms

Among 700 samples, 350 were collected from free range farms and scale farms, respectively. Seven (2.0%) samples were found positive of *T. gondii* DNA in free range farms, whereas no (0%) sample was positive in scale farms (Table 3). The difference was highly significant (*P* < 0.01).

**Table 3:** Contamination of *T. gondii* in the soil collected from different farms

Type of farms	No.of samples examined		Positive number	PCR positive(%)
Free range farms	Feeding zone	175	7	4.00
	motion zone	175	0	0
Scale farms	350		0	0
Total	700		0	1.00

#### Comparison of soil contamination of *T. gondii* between feeding zone and motion zone in free range farms

In free range farms, 175 soil samples were collected respectively from feeding zone and motion zone (Table 3). All of the positive samples (7/175) were from the feeding zone (*P* < 0.01).

#### Soil contamination status of *T. gondii* in different seasons

**Fig. 2:**
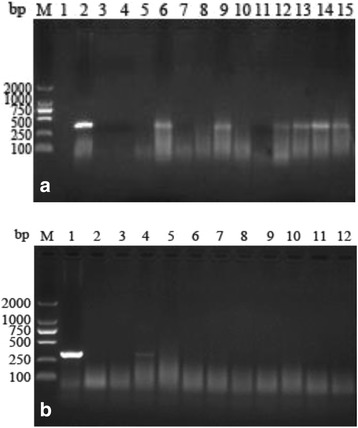
Detection of *T. gondii* in soil collected in autumn and in winter. **a** Lane M: molecular size marker; lane 1: negative control; lane 2: positive control; lanes 3–15: soil samples. **b** Lane M: molecular size marker; lane 1: positive control; lane 2: negative control; lanes 3–12: soil samples

From spring to summer, no positive sample was found by PCR for *T. gondii*. In autumn, *T. gondii* DNA was found in 6 (3.33%) of 180 samples. Out of the 180 samples collected in winter, 1 (0.56%) sample was detected as positive (Fig. 2).

### Detection of *T. gondii* in chickens

In the 350 chickens detected, total 235 (67.14%) were seropositive for *T. gondii.* In the 250 chickens from 5 farms with *T. gondii* positive in soil, 77.60% (194/250) were seropositive. In the 100 chickens from 2 farms with *T. gondii* negative in soil, 41.00% (41/100) were seropositive. On the farm level, all farms had seropositive chickens.

## Discussion

*T. gondii* oocysts in environment samples, especially in soils are getting more and more attention. Studies reported that approximately 1% of cats shed oocysts of *T. gondii* at any given time [1, 23] and they excreted oocysts for a median of 8 days with a total of up to 55 million oocysts per day [1, 23]. The total number of oocysts shed by a single cat varies widely from 3 to 810 million [23]. So it’s necessary to establish a sensitive method to detect the soil contamination status of *T. gondii*. The results from previous studies indicated that PCR methods were expected to be highly specific and to be more efficient to detect low oocyst concentrations [24]. Therefore, in this study PCR assay based on the ITS-1 gene was used to detect *T. gondii* oocysts in soil samples for its high specificity and sensitivity [6]. The results of the current research showed that the detection limit of the method was 50 tachyzoites (equal to 6.25 oocysts) [25] of *T. gondii* in 0.5 g soil. A method previously reported could detect 100–1 000 oocysts in 1 g soil [26]. Compared to this, the PCR method based on the ITS-1 gene established in this study was more sensitive and could be used to detect the *T. gondii* in soil.

Recently, many recombinant surface or secreted antigens have been used successfully either alone or in combination for the detection of specific antibodies due to *T. gondii* infections in humans and animals. A study reported that rGRA7 ELISA showed a high sensitivity and specificity, and rGRA7 can be used as a potential immunogenic antigen for developing immunodiagnostic tools for immunodiagnosis of toxoplasmosis in patients including patients with cancer [27]. GRA8 is a conserved gene of *T. gondii.* Previous studies introduced GRA8 as a marker of acute infection and showed that IgG and IgM ELISA with recombinant GRA8 as antigen was able to differentiate acute from chronic infection [28, 29] and GRA8 ELISA could be used to detect almost all the common strains of *T. gondii* [30, 31]*.* In this study, the truncated GRA8 ELISA method showed that the sGRA8 ELISA could detect specific antibody in all of 20 chickens infected with *T. gondii* 45 DPI, and 95% 60 DPI. The positive rate was still 80% 130 DPI. The serum against common infectious diseases of chicken did not present any cross reactions with this antigen. In the sensitivity experiment, the standard sGRA8 positive serum was diluted to 1: 81 920. These results showed that sGRA8 was validated as a useful antigen and promised a highly sensitive and specific ELISA.

In the past, few studies have been investigated soil contamination with *T. gondii* in scale farms and most studies were focused on free range chicken farms, specifically about the prevalence of *T. gondii* infection in chickens [32–34]. Until now, data on the soil contamination of *T. gondii* in chicken farms from China were missing. So in our study, we collected samples from both free range farms as well as scale farms, and compared the relationship between the status of soil contamination and the infection of *T. gondii* in chickens. The results showed that only free range farms had soil contamination with *T. gondii* and there was no *T. gondii* detected among the samples collected from scale farms. It indicated that the soil contamination in free range farms was higher than that in scale farms. Our results were consistent with that obtained by Jacobs [35] in US. Moreover, we firstly divided the free range farm into feeding zone and motion zone. It was demonstrated that all positive samples were found in feeding zones. It indicated that the feeding zones were the main contamination regions in the farms.

In our study, the seasonal changes of soil contamination were observed. Our results suggested that the *T. gondii* in soil was different from spring to winter. In the four seasons, *T. gondii* was only found in autumn and winter. It was in accord with Dubey’ study [36], that low temperature contributed to the survival of *T.gondii.* This indicated that seasons might have important impacts on the presence of *T. gondii* in soil.

In the serum detection, the average serum positive ratio was 67.14% which was higher than the previously study in our lab (35.15%) [13]. Furthermore, the seropositive rate for *T. gondii* in farms with *T. gondii* positive in soils was significantly higher than the farms without *T. gondii* in soils. It could be concluded that the infections of *T. gondii* in chickens might be affected by the soil contamination. Thus, the soil contamination of *T. gondii* might be an effective indicator of *T. gondii* infection in chickens.

## Conclusion

In conclusion, the results of the present investigation indicated that high seroprevalence of *T. gondii* in chickens was found in the chicken farms with soil contamination by *T. gondii*. The soil contamination in chicken farms all concentrated in the feeding zone of the free range farms, and was influenced by different seasons. Therefore, monitoring of *T. gondii* in soil, combined with good sanitary practices, should be adopted to prevent *T. gondii* infection in chickens for future.

## Additional file

Additional file 1: Multilingual abstracts in the five official working languages of the United Nations. (PDF 807 kb)

## Abbreviations

DPI: Days post-infection
IBDV: Bursal disease virus
ITS-1: Internal transcribed spacer 1
NDV: Newcastle disease virus
PBS: Phosphate buffered saline
SD: Standard deviations
*T. gondii*: *Toxoplasma gondii*
TCA: *T. gondii* circulating antigens
TCAb: *T. gondii* circulating antibodies
