# Supplementary material for: Detection of Toxoplasma gondii in chicken and soil of chicken farms in Nanjing region, China
Source: Infect Dis Poverty. 2017 May 9;6:62. doi: 10.1186/s40249-017-0277-3 (PMC5422862; doi:10.1186/s40249-017-0277-3)
Supplement: Additional file 1: — Multilingual abstracts in the five official working languages of the United Nations. (PDF 807 kb) [file 40249_2017_277_MOESM1_ESM.pdf]

Translation of the abstract into the five official working languages of the United Nations

اكتشاف طفيلي *المقوسة الغوندية* في الدجاج وتربات مزارع الدجاج بمنطقة نانجينغ في الصين

شين- تشاو ليو، يو هى، دينجي هان، جين-تشاو جانغ، كى لي، شواي وانغ، لين-شن زو، روو-فينغ يان، شيانغ-روي لي

الملخص:

الخلفية: من المسلم به بصورة متزايدة أن التربة مصدر مهم لانتقال طفيلي *المقوسة الغوندية* (*T. gondii*). من ثم، كان الهدف من إجراء هذه الدراسة هو التحقق من وجود *المقوسة الغوندية* في التربة بالإضافة إلى فهم العلاقة بين تلوث التربة بالطفيلي وإصابة الدجاج به.

المنهج: تم وضع وتطبيق منهج تفاعل البوليميراز المتسلسل بناءً على الفاصل الداخلي المنسوخ الأول للحمض النووي الديوكسي للجين المحفوظ لطفيلي *المقوسة الغوندية* (ITS-1) باعتباره الجين المستهدف، ومنهج المقايسة الامتصاصية المناعية للإنزيم المرتبط (sGRA8-ELISA) باستخدام البروتين المؤتلف لجين GRA8 المقتطع من طفيلي *المقوسة الغوندية* باعتباره مستضد. بدءًا من أبريل/ نيسان عام 2013 وحتى مارس/ آذار عام 2014، تم أخذ ما يصل إلى 700 عينة من تربات عدة مواقع بثلاثين مزرعة مصنفة باعتبارها مزارع مفتوحة ومزارع تلقيح بمنطقة نانجينغ بمقاطعة جيانغسو في الصين في مواسم مختلفة. علاوةً على ذلك، تم أخذ ما يصل إلى 350 مصلاً من الدجاج من المزارع المفتوحة لتحديد وجود أجسام مضادة لطفيلي *المقوسة الغوندية* بها من عدمه باستخدام منهج sGRA8-ELISA.

النتائج: أظهرت نتائج الاختبارات المصلية وجود أجسام مضادة في 194 عينة من أصل 250 عينة (67.14 %) مأخوذة من مزارع جاءت نتائج وجود طفيلي *المقوسة الغوندية* بتربتها إيجابية، و41 عينة من أصل مائة عينة مأخوذة من مزارع جاءت نتائج وجود الطفيلي بها سلبية (41.00 %) (*القيمة الاحتمالية* < 0.01). أظهر منهج اكتشاف تفاعل البوليميراز المتسلسل في عينات التربة وجود طفيلي *المقوسة الغوندية* في 7 عينات (2.0 %) من أصل 350 عينة مأخوذة من منطقة العلف في المزارع المفتوحة، وعدم وجوده في أية عينة من العينات المأخوذة من مزارع التلقيح. أما بالنسبة للاكتشافات الموسمية، فقد وُجد طفيلي *المقوسة الغوندية* في 6 (3.33 %) عينات أُخذت في فصل الخريف وفي عينة واحدة فقط (0.56 %) أُخذت في فصل الشتاء.

الاستنتاجات: بينت نتائج الأبحاث أن معدل تلوث تربة المزارع المفتوحة بطفيلي *المقوسة الغوندية* أعلى من معدل تلوث تربة مزارع التلقيح به، وأن معدل الانتشار المصلي للطفيلي في دجاج المزرعة ذات التربة الملوثة بالطفيلي أعلى من معدل انتشاره في المزرعة ذات التربة غير الملوثة؛ وبالتالي، قد يكون تلوث التربة مؤشرًا فعالاً على إصابة الدجاج بطفيلي *المقوسة الغوندية*.

Translated from English version into Arabic by Heba Kandel, through

TRANSLATORS
WITHOUT BORDERS

中国南京地区鸡弓形虫感染和鸡场土壤弓形虫污染情况检测

刘欣超，何瑜，韩登阁，张振超，李珂，王帅，徐立新，严若峰，李祥瑞

摘要：

引言：近来，土壤被认为是弓形虫传播的一种重要途径。本文的目的是研究土壤弓形虫的污染情况，并掌握土壤污染情况和鸡弓形虫感染之间的关系。

方法：建立以弓形虫保守基因 ITS-1 为靶基因的 PCR 方法和以截短的重组 GRA8 蛋白为抗原的 sGRA8-ELISA 方法。从 2013 年 4 月到 2014 年 3 月，在江苏南京 30 家散养鸡场和集约化养殖场的多个地点采集 700 份土壤样品，并分不同季节进行采样。此外，在散养鸡场采集 350 份鸡血清，用 sGRA8-ELISA 检测鸡弓形虫感染情况。

结果：血清学检测结果显示：从存在土壤弓形虫污染的鸡场采集的 250 份血清，有 194 份为弓形虫阳性（67.14%），无土壤弓形虫污染的鸡场采集的 100 份血清，有 41 份为弓形虫阳性（41%）（P< 0.01）。PCR 检测土壤样品的结果显示，采自散养鸡场的 350 份土壤样品中，有 7 份采自饲养区的样品为弓形虫阳性。而采自集约化养殖场的 350 份样品未检出弓形虫。在不同季节的检测中，秋天有 6 份土壤弓形虫阳性，冬天有 1 份土壤弓形虫阳性。
结论：结果显示散养鸡场土壤的弓形虫污染情况要高于集约化养殖场，并且存在土壤弓形虫污染的鸡场，鸡血清学检测阳性率要高于无土壤弓形虫污染的鸡场。所以，土壤的污染的情况可以提示鸡弓形虫感染的情况。

Translated from English version into Chinese by Xin-Chao Liu

Détection du *Toxoplasma gondii* chez des poules et dans le sol d'élevages de poules dans la région de Nanjing, Chine

Xin-Chao Liu, Yu He, Dengge Han, Zhen-Chao Zhang, Ke Li, Shuai Wang, Li-Xin Xu, Ruo-Feng Yan, Xiang-Rui Li

Résumé

Contexte : le sol est de plus en plus reconnu comme constituant une source importante dans la transmission du *Toxoplasma gondii* (*T. gondii*). L'objectif de la présente étude consistait à détecter la présence du *T. gondii* dans le sol et à mieux comprendre les relations entre la contamination du sol et les infections de poules.
Méthodes : une méthode PCR reposant sur l'espaceur interne transcrit 1 du gène conservé du *T. gondii* (ITS-1) à titre de gène cible et la méthode ELISA (sGRA8-ELISA) reposant sur l'utilisation de la protéine recombinante du gène GRA8 raccourci du *T. gondii* à titre d'antigène ont été développées et appliquées. D'avril 2013 à mars 2014, 700 échantillons de sol ont été collectés sur différents sites auprès de trente élevages différents, de plein air et en batterie, à Nanjing, Chine, à différentes saisons. De plus, 350 sérums de poulet ont été collectés auprès d'élevages en plein air afin de déterminer la présence d'anticorps dirigés contre le *T. gondii* à l'aide de la méthode sGRA8-ELISA.
Résultats : d'après les résultats sérologiques, des anticorps ont été détectés dans 194 échantillons de sol fermier sur 250 (67,14 %) positifs au *T. gondii* et 41 échantillons de sol fermier sur 100 (41,00 %) négatifs au *T. gondii* (p<0,01 %). La détection par PCR d'échantillons de sol a démontré que 7 (2,0 %) des 350 échantillons collectés dans la zone de nourrissage d'élevages en plein air s'étaient avérés positifs au *T. gondii*, tandis qu'aucun échantillon positif n'avait été détecté au sein d'élevages en batterie. Dans le cadre des détections saisonnières, le *T. gondii* a été détecté dans 6 (3,33 %) des échantillons collectés à l'automne et dans 1 (0,56 %) des échantillons collectés en hiver.
Conclusions : les résultats ont indiqué que le taux de contamination du *T. gondii* dans le sol d'élevages en plein air était plus élevé que dans les élevages en batterie et que la séroprévalence du *T. gondii* chez les poules vivant dans un élevage dont le sol est contaminé était plus élevée que chez les poules vivant dans un élevage sans contamination du sol. Le degré de contamination du sol est susceptible de constituer un indicateur efficace de l'infection par le *T. gondii* chez les poules.

Translated from English version into French by eric ragu, through

Обнаружение *Toxoplasma gondii* в курицу и почвы куриных ферм в регионе Нанкин Китая

Xin-Chao Liu, Yu He, Dengge Han, Zhen-Chao Zhang, Ke Li, Shuai Wang, Li-Xin Xu, Ruo-Feng Yan, Xiang-Rui Li

Реферат

Фон: Почва все более широко признается в качестве важного источника в передаче *Toxoplasma gondii* (*T.* gondii). Целью данного исследования было изучить наличие T. gondii в почве и понять взаимосвязь между загрязнением почвы и курицей инфекций.

Метод: PCR основан на *T. gondii*-conserved внутренние ген, транскрибируемый спейсер 1 (ITS-1) в качестве целевого Гена и метод ELISA (sGRA8-ELISA) с использованием рекомбинантного белка укороченных генов GRA8 при *T. gondii* в качестве антигена были разработаны и применены. С апреля 2013 по март 2014 года в общей сложности 700 почвы, отобранных на различных объектах, расположенных в тридцати хозяйств классифицируются как бесплатно ферма диапазона и масштаба фермы в Нанкине, Цзянсу Китая, в разных сезонах. Кроме того, были собраны из свободного ферм, чтобы определить наличие антител против *T. gondii* в общей сложности 350 сыворотках крови кур с помощью sGRA8-ELISA.

Результаты: Серологические результаты показали, что антитела были найдены в 194 из 250 (67.14%) образцов из хозяйств с *T. gondii* положительное в почве и 41 из 100 образцов из хозяйств с *T. gondii* отрицательный в почве (41.00%)($p < 0,01$). Обнаружение методом PCR образцов грунта показало, что 7 (2.0%) из 350 образцов, взятых из зоны кормления при свободном выгуле хозяйств были выявлены положительные *T. gondii* а не проба оказалась положительной в фермерских хозяйствах. В сезонном объекте, *T. gondii* была обнаружена в 6 (3.33%) образцах, собранных осенью и 1 (0.56%), собранных земной.

Заключение: Результаты показали, что заражение *T. gondii* в почве в свободном ряде хозяйств было выше, чем в фермерских хозяйствах, а число ВИЧ-инфицированных *T. gondii* в курах в хозяйстве с плотностью загрязнения почвы был выше, чем без загрязнения почв. Загрязнение почвы может быть эффективным индикатором инфекция *T. gondii* в курах.

Translated from English version into Russian by Hao-Qi Zhang

Detección de Toxoplasma gondii en pollos y suelo de granjas de pollos en la región de Nanjing, China

Xin-Chao Liu, Yu He, Dengge Han, Zhen-Chao Zhang, Ke Li, Shuai Wang, Li-Xin Xu, Ruo-Feng Yan, Xiang-Rui Li

Resumen

Antecedentes: El suelo es reconocido, cada vez más, como una fuente importante en la transmisión de *Toxoplasma gondii (T. gondii)*. El objetivo de este estudio fue investigar la presencia de *T. gondii* en el suelo y captar la relación entre la contaminación del suelo y las infecciones de los pollos.

Métodos: Se desarrollaron y aplicaron el método de PCR basado en el *T. gondii*-gen conservado espaciador transcrito interno 1 (ITS-1) como gen objetivo y el método ELISA (sGRA8-ELISA) utilizando la proteína recombinante del gen GRA8 acortado de *T. gondii* como antígeno. De abril de 2013 a marzo de 2014, se recolectaron, en diferentes estaciones, un total de 700 muestras de suelo en varios sitios ubicados en treinta granjas clasificadas como "de campo libre" (donde los animales comen libremente en el campo) y "a escala", en Nanjing, Jiangsu, China. Adicionalmente, se recogieron 350 sueros de pollos de granjas de campo libre para determinar la presencia de anticuerpos contra *T. gondii* utilizando sGRA8-ELISA.

Resultados: Los resultados serológicos mostraron anticuerpos en 194 de las 250 muestras (67,14%) de granjas con *T. gondii* positivo en el suelo y en 41 de 100 muestras de granjas con *T. gondii* negativo en el suelo (41,00%) ($P < 0,01$). La detección PCR de muestras de suelo mostró que 7 (2,0%) de 350 muestras recogidas de la zona de alimentación en las granjas de campo libre dieron positivas de *T. gondii*, mientras que ninguna muestra fue positiva en las granjas a escala. En las detecciones estacionales, se encontró *T. gondii* en 6 (3.33%) muestras recogidas en otoño y en 1 (0.56%) recogida en invierno.

Conclusiones: Los resultados indicaron que la contaminación de *T. gondii* en el suelo en las granjas de campo libre fue mayor que en las granjas a escala y la seroprevalencia de *T. gondii* en los pollos de la granja con contaminación del suelo fue mayor que la de la granja sin contaminación del suelo. La contaminación del suelo podría ser un indicador efectivo de la infección por *T. gondii* en los pollos.

Translated from English version into Spanish by patriciacassoni, through
